# Development of Novel Faster-Dissolving Microneedle Patches for Transcutaneous Vaccine Delivery

**DOI:** 10.3390/pharmaceutics9030027

**Published:** 2017-08-03

**Authors:** Akihiko Ono, Sayami Ito, Shun Sakagami, Hideo Asada, Mio Saito, Ying-Shu Quan, Fumio Kamiyama, Sachiko Hirobe, Naoki Okada

**Affiliations:** 1 Project for Vaccine and Immune Regulation, Graduate School of Pharmaceutical Sciences, Osaka University, 1-6 Yamadaoka, Suita, Osaka 565-0871, Japan; ono-ak@phs.osaka-u.ac.jp (A.O.); ito-sa@phs.osaka-u.ac.jp (S.I.); sakagami-s@phs.osaka-u.ac.jp (S.S.); 2 Laboratory of Biotechnology and Therapeutics, Graduate School of Pharmaceutical Sciences, Osaka University, 1-6 Yamadaoka, Suita, Osaka 565-0871, Japan; sachi-be@phs.osaka-u.ac.jp; 3 Department of Dermatology, Nara Medical University, 840 Shin-cho, Kashihara, Nara 634-8522, Japan; asadah@naramed-u.ac.jp; 4 CosMED Pharmaceutical Co. Ltd., 32 Higashikujokawanishi-cho, Minami-ku, Kyoto 601-8014, Japan; saito@cosmed-pharm.co.jp (M.S.); quan@cosmed-pharm.co.jp (Y.-S.Q.); kamiyama@cosmed-pharm.co.jp (F.K.); 5 Laboratory of Vaccine and Immune Regulation, Graduate School of Pharmaceutical Sciences, Osaka University, 1-6 Yamadaoka, Suita, Osaka 565-0871, Japan

**Keywords:** transcutaneous vaccine, faster-dissolving microneedle, carboxymethylcellulose, hyaluronan, clinical research, microneedle-dissolution kinetics in the skin, microneedle failure force

## Abstract

Microneedle (MN) patches are promising for transcutaneous vaccination because they enable vaccine antigens to physically penetrate the stratum corneum via low-invasive skin puncturing, and to be effectively delivered to antigen-presenting cells in the skin. In second-generation MN patches, the dissolving MNs release the loaded vaccine antigen into the skin. To shorten skin application time for clinical practice, this study aims to develop novel faster-dissolving MNs. We designed two types of MNs made from a single thickening agent, carboxymethylcellulose (CMC) or hyaluronan (HN). Both CMC-MN and HN-MN completely dissolved in rat skin after a 5-min application. In pre-clinical studies, both MNs could demonstrably increase antigen-specific IgG levels after vaccination and prolong antigen deposition compared with conventional injections, and deliver antigens into resected human dermal tissue. In clinical research, we demonstrated that both MNs could reliably and safely puncture human skin without any significant skin irritation from transepidermal water loss measurements and ICDRG (International Contact Dermatitis Research Group) evaluation results.

## 1. Introduction

Antigen-presenting cells (APCs) populate the skin to defend against foreign substances. Langerhans cells (LCs) in living epidermis and dermal dendritic cells (dDCs) in dermis are potent APCs and contribute to acquired immune responses [1]. Vaccination via subcutaneous injection (SCI) delivers vaccine antigens into subcutaneous tissue that is almost devoid of these immunocompetent cells. On the other hand, transcutaneous immunization (TCI) can represent an alternative vaccination method to conventional injections because the transcutaneous vaccination delivery system is used for targeting these immunocompetent cells. However, developing transcutaneous vaccine delivery systems is difficult because the stratum corneum, which is the outermost layer of the skin, functioning as a potent physical barrier separating the internal body from the external environment, limits material permeation. A microneedle (MN) patch, composed of micron needles less than 1 mm, can efficiently deliver vaccine antigens to skin-resident APCs under the stratum corneum because vaccine antigens can physically penetrate the stratum corneum through the micropores painlessly formed by the MN patch application to the skin. Therefore, TCI using MN patches is a promising approach for obtaining more effective vaccine efficacy compared with SCI because skin-resident APCs can effectively induce acquired immune responses. Hence, many studies are being undertaken worldwide toward the development of transcutaneous vaccine delivery systems with MN patches [2,3,4,5,6,7].

MNs are categorized into five types: (1) solid MN; (2) hollow MN; (3) coated MN; (4) dissolving MN; and (5) swelling MN. The first three types are commonly considered the first generation of MNs, while dissolving MN and swelling MN represent the second generation type [2,8,9,10,11,12,13]. The first generation MNs present a potential risk of serious adverse effects caused by broken MNs remaining in the skin after application because non-bioabsorbable materials such as silicon and metal are used as MN constituents for their stiffness and molding properties. The second generation MNs, dissolving MNs, use biogenic substances or biocompatible materials such as hyaluronan (HN), chondroitin sulfate, and carboxymethylcellulose (CMC) as constituents. In addition, dissolving MNs can reliably deliver the vaccine antigen loaded inside the MNs into the skin because the punctured MNs themselves dissolve in the skin after application. Hence, dissolving MNs are the most promising tools for clinical use because they can effectively overcome any safety issues of skin irritation caused by broken MNs. In particular, there are no potential future health concerns with dissolving sugar-based MNs because the dissolved materials are completely eliminated from the body. Our MicroHyara^®^ (MH), a dissolving MN patch, was demonstrated to be clinically useful for transcutaneous vaccine delivery and applicable as an alternative vaccination method to conventional injections in clinical research [14,15]. We reported that MH can safely and reliably puncture human skin and trivalent influenza hemagglutinin (HA) antigen-loaded MH can induce antigen-specific immune responses against each HA antigen with neither local nor systemic adverse effects after human skin application.

The current MH patch needs to be applied to human skin for 6 h to reliably deliver the vaccine antigen into the skin, due to the slow dissolution of the MNs in the skin [14,15]. In the current clinical practice in Japan, to check serious adverse events such as allergic and anaphylactic reactions caused by the administered vaccine antigen, it is recommended that vaccinated individuals should be kept under observation in the hospital for at least 30 min after vaccination. Nokleby reported that it was commonly recommended that inoculated individuals should be kept under observation for 15 to 20 min after vaccination because allergy and anaphylactic reactions that might be caused by the administered vaccine antigens appear within 1 h after vaccination [16]. Therefore, in the case of transcutaneous vaccination using a dissolving MN patch, the time required for MN dissolution in the skin after skin application should be less than 30 min considering the total clinical operation time for the healthcare provider.

In the present study, we aimed to design novel faster-dissolving MN patches with MNs dissolving in the skin within 30 min after application, to promote the practical use of dissolving MN patches. To this end, we designed two prototypes of faster-dissolving MNs with the safety and reliable skin puncturability of the original MH. The prototype is made out of a single thickening agent commonly used for dissolving MN and we integrated fundamental findings to optimize the MN formulation with modifications. We selected two thickening agents to prepare the prototype MNs: CMC and HN, which is the main constituent of MH. CMC is a highly safe hydrophilic polymer and an excipient commonly used in pharmaceutical products. It is generally used as a thickening and suspending agent in SCI and as an aqueous gel base in transdermal poultice patches. Hence, examples of formulation design of dissolving MN using CMC have been reported [17,18,19,20,21,22]. However, there were no reports about the required application time to human skin and the time required for MN dissolution in human skin after application for CMC. In this study, we focused on the physicochemical properties and safety of CMC, and assessed the CMC-based formulation (CMC-MN) as well as the HN-based formulation (HN-MN) to confirm the usefulness of CMC for rapid MN dissolution in human skin.

## 2. Materials and Methods

### 2.1. Animals

Wistar rats (six- to eight-week-old, female) were purchased from Japan SLC (Hamamatsu, Japan) and were housed at the Osaka University animal facility. All animal studies were conducted in accordance with the guidelines provided by the Animal Care and Use Committee of Osaka University.

### 2.2. Fabrication of Faster-Dissolving MN Patches and Measurement of Fracture Force for MN

CMC-MN and HN-MN were made from CMC (Daicel FineChem, Tokyo, Japan) and HN (Kikkoman Biochemifa, Tokyo, Japan), respectively. Both MN patches were aseptically fabricated using a micromolding technology. CMC or HN was dissolved in distilled water and each aqueous solution was casted onto micromolds. Subsequently, MN patches were obtained from micromolds after drying in a desiccator at room temperature. Faster-dissolving MN patches loaded with fluorescein-labeled ovalbumin (F-OVA; Molecular Probes, Eugene, OR, USA) or EndoGrade^®^ Ovalbumin (OVA; Hyglos GmbH, Bernried, Germany) were fabricated in the same manner, using an aqueous solution of the dissolved thickening agent and F-OVA or OVA. The prepared MN patches were stored in an aluminum laminate PET bag under refrigeration until they were used in the experiments.

Mechanical failure tests for MN patches were performed with TA-XT plus texture analyzer (Stable Micro Systems, Godalming, UK), as described previously [23,24]. An MN patch was horizontally mounted in a test station with the needles placed vertically in it. Axial force was then applied using a flathead 0.5-mm-diameter stainless steel cylinder to move the cylinder at a rate of 0.1 mm/second on the MNs. The required force for mechanical MN fracture was measured and the necessary fracture force per needle (N/needle) was calculated by dividing the measured fracture force by the number of fractured MNs.

### 2.3. Analysis of MN-Dissolution Kinetics after Puncturing the Skin

The faster-dissolving MN patch for placebo was applied to the hair-removed back skin of Wistar rats using a handheld spring applicator [15,23,24]. After skin application for 1, 2, 5, or 10 min, the MN patch was removed from the skin and the punctured MNs were immediately observed with a stereoscopic microscope (VHX-1000; KEYENCE, Osaka, Japan).

### 2.4. In Vivo Fluorescence Imaging for Antigen Deposition Assessment

The F-OVA (36 μg protein)-loaded MN patch was applied to the hair-removed back skin of Wistar rats using a handheld spring applicator [15,23,24]. After skin application of a MN patch for 5 min, fluorescence images of the application site were obtained using the CRi Maestro EX in vivo imaging system (Cambridge Research and Instrumentation, Woburn, MA, USA) following a predefined schedule, as described previously [24]. In the comparison group, phosphate-buffered saline (PBS) containing F-OVA (36 μg/50 μL) was intradermally (ID) administered into the back skin of rats. Fluorescein images were captured at an exposure time of 200 ms and the spectral resolution for all imaging was 10 nm. Measurements of integrated fluorescence intensity for the MN-application or injection site were performed using Maestro version 2.10 software (Cambridge Research and Instrumentation, Cambridge, MA, USA), and percentage of the residual fluorescence intensity at each time point relative to 0 h was calculated.

### 2.5. Vaccination and Measurement of OVA-Specific IgG Titers

Faster-dissolving MN patches loaded with 10 μg of OVA were applied to the dehaired back skin of Wistar rats for 10 min or 4 h using a handheld spring applicator [15,23,24]. Vaccination was repeated three times at two-week intervals. The comparison group was subcutaneously immunized with PBS containing OVA (OVA/PBS solution). The same dose of OVA/PBS solution (10 μg/50 μL) or the tenfold dose of OVA/PBS solution (100 μg/50 μL) was repeatedly administered three times at two-week intervals.

OVA-specific IgG titers in sera were determined by ELISA. OVA-coated ELISA plates were blocked, and then half-fold serial dilutions of sera were added and incubated for 2 h at 25 °C. HRP-conjugated goat anti-rat IgG (Southern Biotech, Birmingham, AL, USA) was used for the detection of rat OVA-specific IgG. The OVA-specific IgG titers were expressed as the reciprocal log_2_ titer of the highest dilution that generated 0.1 absorbance units after subtracting the absorbance of preimmune sera.

### 2.6. Fluorescence Imaging for MN Insertion into Excised Human Skin

After review and approval by the Institutional Review Board for Clinical Research at Osaka University Hospital, surplus resected human dermal tissue derived from surgery was kindly provided by a volunteer. F-OVA (36 μg protein)-loaded faster-dissolving MN patch was applied to the surgically excised human dermal tissue using a handheld spring applicator and left in place for 1 h [15,23,24]. After removal of the patch, the skin was frozen in OCT compound (Sakura Finetechnical, Tokyo, Japan) and cut into 8-μm-thick sections using a cryostat, as described previously [23,24]. Histological examination of the skin was performed on frozen sections that were mounted with Prolong Gold antifade reagent with DAPI (Invitrogen, Carlsbad, CA, USA) and then photographed using fluorescence microscopy (BZ-8000; KEYENCE, Osaka, Japan).

### 2.7. Transepidermal Water Loss (TEWL) Measurement and Human Safety Study after Placebo MN Patch Applications in Clinical Research

Nineteen healthy volunteers (aged 19–54; 14 male and five female, Japanese) were enrolled in the study. Informed consent was obtained from all volunteers before enrollment. All clinical procedures were approved by the Ethics Committee of Nara Medical University. Both novel faster-dissolving MN patches as placebo were applied to the lateral upper arms of 19 healthy volunteers using a spring-type applicator. After skin application for 30, 60 or 120 min, MN patches were removed from the skin and the punctured MN patches were immediately observed under a stereoscopic microscope (VHX-1000; KEYENCE, Osaka, Japan).

To evaluate the degree of skin barrier dysfunction, TEWL at the application site was measured immediately after removal of the MN patch left for 30 min on the skin, using a Closed-chamber type Mobile Tewameter (VAPO SCAN AS-VT100RS; Asahi Biomed, Tokyo, Japan). Local adverse responses at the skin application site were investigated on the second and seventh days after MN patch application. The degree of skin irritation observed was scored in accordance with the International Contact Dermatitis Research Group (ICDRG) scoring system, as shown in Table 1.

### 2.8. Statistical Analysis

Data are expressed as mean ± SEM of results from four or six rats. If the OVA-specific IgG titers in sera might be estimated less than 6, the value was expressed as 6. For multiple comparison, a one-way analysis of variance (ANOVA) was carried out, followed by Bonferroni–Dunn test as a post hoc test (Statcel version 2.0 software, OMS Publishing, Tokorozawa, Japan). In all cases, *p* < 0.05 and *p* < 0.01 were considered statistically significant and highly significant, respectively.

For TEWL measurements in the clinical research, the statistical significance of difference between two MN groups was analyzed. For multiple comparisons, ANOVA was carried out, followed by the Bonferroni–Dunn test as a post hoc test (Statcel version 2.0 software, OMS Publishing, Tokorozawa, Japan). In all cases, *p* < 0.05 and *p* < 0.01 were considered statistically significant and highly significant, respectively.

## 3. Results

### 3.1. Pharmaceutical Characteristics and MN-Dissolution Kinetics of Faster-Dissolving MN Patches

**Figure 1:**
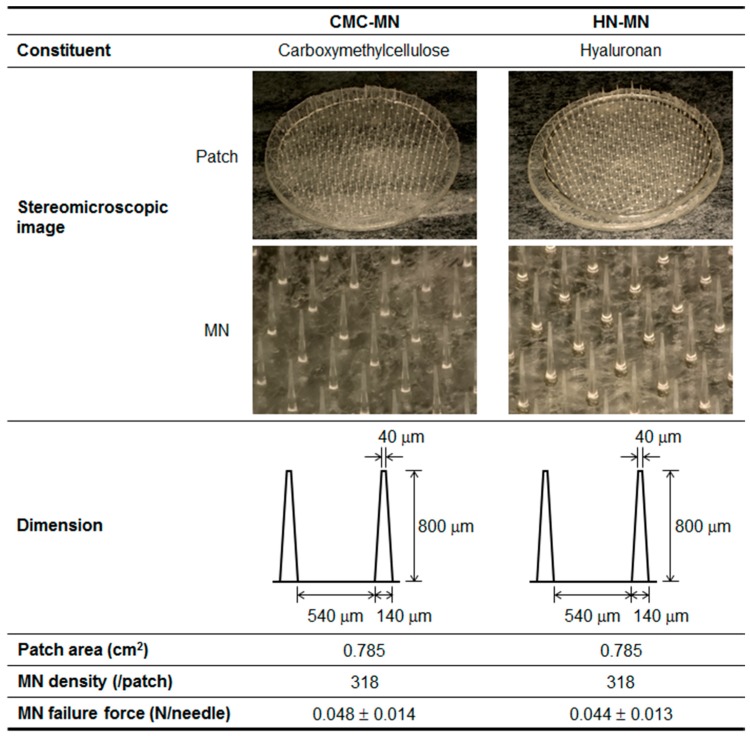
Pharmaceutical characteristics of novel faster-dissolving MN patches. Data of MN failure forces are expressed as mean ± SD of results from four measurements.

Pharmaceutical characteristics of two types of novel faster-dissolving MN patches, made with CMC or HN, are summarized in Figure 1. Both MNs were identical in dimension and had the following pharmaceutical properties—length: 800 μm, width at tip: 40 μm, width at basement: 140 μm, needle-to-needle spacing: 540 μm, and MN density: 318 needles/0.785 cm^2^. The MN failure force, which is a critical quality attribute for reliable skin puncturability of MNs, of CMC-MN and HN-MN was 0.048 and 0.044 N/needle, respectively.

**Figure 2:**
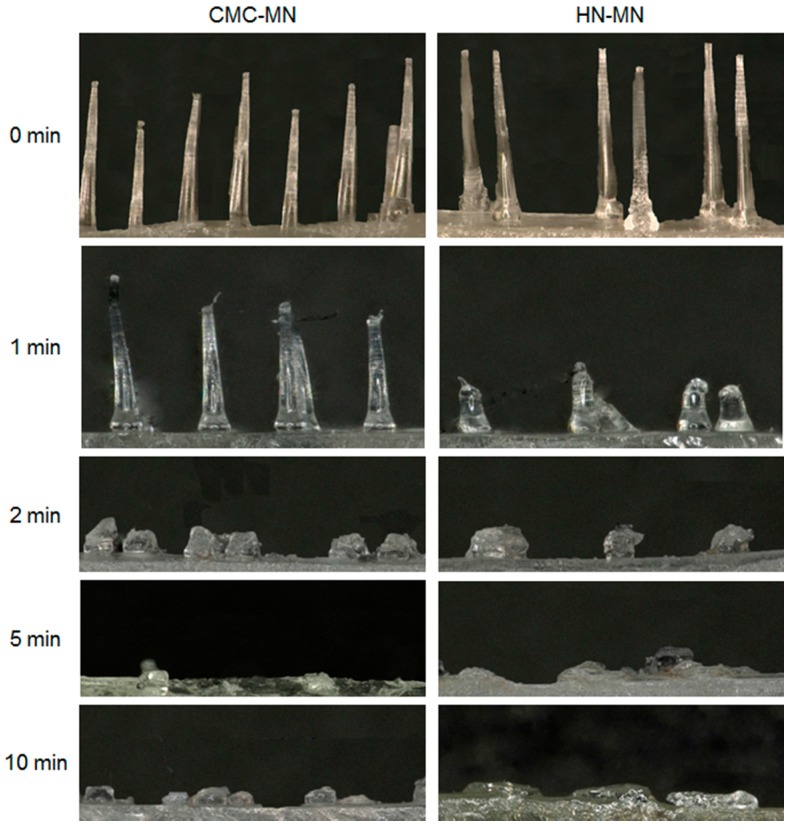
MN-dissolution kinetics of faster-dissolving MNs after patch applications to rat skin. Placebo CMC-MN and HN-MN patches were applied to the back skin of Wistar rats for 1, 2, 5, or 10 min. After removal of MN patches, the remaining MNs on each CMC-MN and HN-MN patch were photographed using a stereoscopic microscope.

To test the faster-dissolving MN proof of concept, we evaluated the dissolution kinetics of MNs after MN patch application to rat skin (Figure 2). For both MNs, the MN lengths were reduced not less than 70% after 2 min of application to rat skin and the MNs were almost completely dissolved after 5 min of application. Based on the proof of concept in rats, both novel MNs are promising with faster-dissolving MNs for human skin application.

### 3.2. Localization and Deposition of Antigen Delivery by the MN Patches

**Figure 3:**
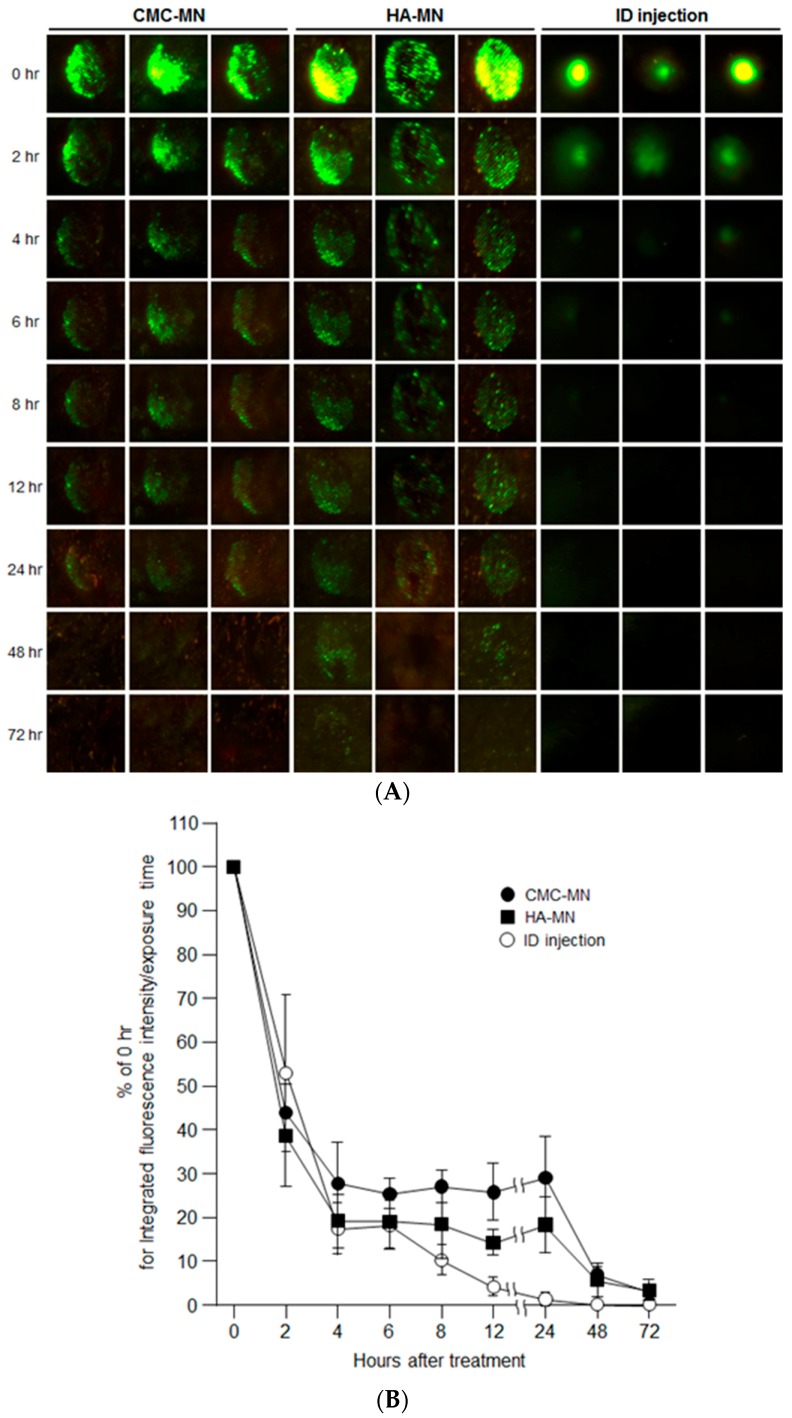
Time course of antigen deposition at the skin administration site after F-OVA administration to rats via an MN patch or ID injection. (**A**) Representative in vivo fluorescence imaging for antigen deposition from three rats for each group, transcutaneous administration of CMC-MN and HN-MN patches, and ID injection; (**B**) Time course profiles of the residual fluorescence intensity at the administration site after F-OVA-loaded MN patch applications or ID injection. The residual fluorescence intensity at the administration site was defined as % of the initial fluorescence intensity at 0 h. Data are expressed as mean ± SEM of results from three rats.

After providing rapid MN dissolution of our novel MN patches, we next assessed the deposition of transcutaneous administered antigen in rat skin over time by in vivo fluorescence imaging after F-OVA administration using MN patches compared with ID injection (Figure 3). Each green fluorescence spot remained for up to 24 h for CMC-MN and 48 h for HN-MN after skin application, whereas a slight green spot remained for up to 4 h after ID injection. The time course profile of the percentage of fluorescence intensity relative to 0 h for the administration site also revealed that faster-dissolving MN patch administration resulted in prolonged antigen deposition compared with ID injection. The percentage of fluorescence intensity at the ID administration site markedly decreased after 4 h of administration and was less than 5% at 12 h after administration. On the other hand, up to 24 h after administration, the percentage of fluorescence intensity at the administration site via MN patch was maintained at more than 20% for CMC-MN and more than 10% for HN-MN.

Hence, the in vivo fluorescence imaging results revealed that the transcutaneous administration via our faster-dissolving MN patches could extend antigen deposition in the skin compared with ID injections.

### 3.3. In Vivo Antigen-Specific Immune Response Induction using OVA-Loaded MN Patches

We next examined the induction of antigen-specific immune responses in rats using OVA-loaded MN patches to confirm the efficacy of transcutaneous vaccination in vivo.

**Figure 4:**
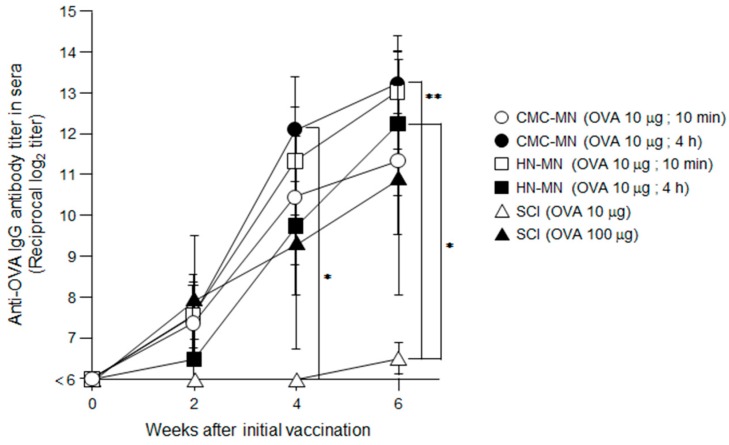
OVA-specific antibody titers induced by TCI using OVA-loaded, faster-dissolving MN patches. OVA (10 μg)-loaded faster-dissolving MN patches were applied to the back skin of Wistar rats for 10 min or 4 h three times at two-week intervals. As controls, each OVA solution in the same dose (10 μg/50 μL) and in the tenfold dose (100 μg/50 μL) was subcutaneously administered three times at two-week intervals. Sera collected from these rats were assayed for the IgG titer specific for OVA by ELISA. Data are expressed as mean ± SEM of results from four or six rats. ∗ *p* < 0.05 vs. SCI (10 μg), ∗∗ *p* < 0.01 vs. SCI (10 μg).

The induction of OVA-specific IgG titers in sera were detected after the first immunization using MN patches in rats (Figure 4). In addition, the repeated TCI through the second and third administration by MN patches increased the OVA-specific IgG titers. Profiles of increase of OVA-specific IgG titers by HN-MN application and CMC-MN application were equivalent, and the vaccine effects by a 10-min application in both MN groups was comparable to those by 4-h application. These results revealed that our novel faster-dissolving MN patches can demonstrate sufficient ability for transcutaneous vaccine delivery with just a 10-min application. In comparison with SCI, the induction of OVA-specific IgG titers in sera were first detected after the third immunization at the same dose of TCI than for MN patches (10 μg/rat). The production of OVA-specific IgG tiers in CMC-MN group by 4-h application after the second and third immunization were significantly greater than those in SCI group at the dose of 10 μg/rat. In HN-MN group by 4-h application, the third immunization significantly increased OVA-specific IgG titers greater than that in SCI group at the dose of 10 μg/rat. Additionally, the elevation profile of the induced OVA-specific IgG titers in sera at the dose of 100 μg/rat was similar to those of TCI with MN patches at a dose of 10 μg/rat.

These results confirm that our novel faster-dissolving MN patches are effective TCI devices for vaccination because they could induce in vivo antigen-specific immune responses, a fundamental attribute of a transcutaneous vaccine delivery device. Furthermore, the results of antibody production after the first immunization demonstrated that the TCI with faster-dissolving MN patches could induce antigen-specific immune responses more strongly than conventional SCI.

### 3.4. Antigen Delivery, Dissolution Kinetics of MNs, Skin Puncturability, and Safety of Faster-Dissolving MN Patches in Humans

**Figure 5:**
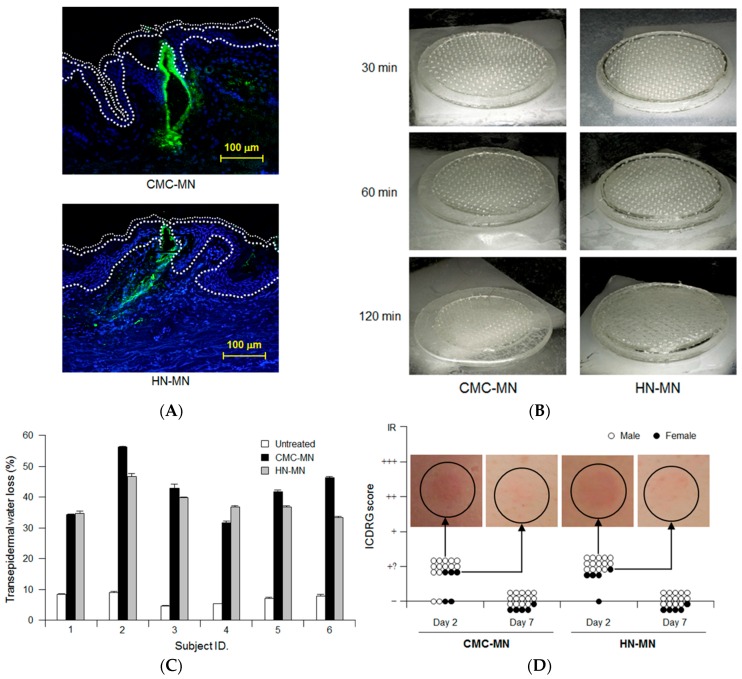
Antigen delivery images, dissolution of MNs, skin puncturability and safety using faster-dissolving MN patches on human skin. (**A**) F-OVA-loaded MN patches were applied to the resected human dermal tissues. In the fluorescence images, the area between the top line and the middle line represents the stratum corneum, the area between the middle line and the bottom line represents the living epidermis, and the dermis is located under the bottom line. Green and blue fluorescence indicate F-OVA and nucleus (DAPI), respectively; (**B**) Placebo CMC-MN and HN-MN patches were each applied to the skin of upper outer arm of 19 healthy volunteers (14 male and five female) for the indicated times. After removal of MN patches, the remaining MNs on each CMC-MN and HN-MN patch were photographed using a stereoscopic microscope; (**C**) After MN patch applications of 30 min, TEWL at the application site was measured immediately after MN patch removal. Data are expressed as mean ± SD of results from three measurements; (**D**) Skin irritation caused by application of MN patches was assessed in accordance with the ICDRG score. Each plot expresses the score of an individual subject. Four photographs show the site judged as +? “doubtful reaction; faint erythema only.”

We evaluated the dissolution kinetics of MNs, the skin puncturability and safety of our novel faster-dissolving MN patches in clinical research as well as the antigen delivery in resected human skin. Prior to clinical research, we fundamentally assessed the antigen delivery abilities of faster-dissolving MN patches by applying F-OVA-loaded MN patches to resected human dermal tissue (Figure 5A). Fluorescence images of histological sections demonstrated that the fluorescence derived from F-OVA (green spot) was observed in both living epidermis and dermis. The results suggested that our novel faster-dissolving MN patches could reliably deliver the loaded vaccine antigens into human skin with a patch application.

In clinical research, we first analyzed the dissolution kinetics of MNs by visual confirmation of MN patch appearance after human skin application. Figure 5B shows each representative result of MN patch appearance after 30 min, 60 min, and 120 min of human skin application, respectively. The degrees of MN dissolution in both MN patches were approximately 50% and 80% reduction in length after 30 min and 60 min, respectively. The MNs in both MN patches completely dissolved after 120 min of human skin application.

We next assessed human skin puncturabilities of our novel MN patches by measuring TEWL at the skin application site (Figure 5C). TEWL, an index of skin barrier dysfunction, has been widely used to assess the human skin puncturability of MN patches because TEWL increment at the MN patch application site suggests that micropores in the human stratum corneum were formed by insertion of MNs into the skin [25]. Both TEWLs for the MN patch application sites were higher than those for untreated sites in all the investigated subjects (6 subjects). In addition, there was no significant difference of TEWLs for the MN patch application sites between CMC-MN and HN-MN. Therefore, both MN patches had sufficient ability to insert MNs into human skin and the human skin puncturability for CMC-MN was similar to that for HN-MN.

We carefully monitored adverse events at the site of MN patch application over time and assessed the observation according to ICDRG scores. Erythemas were observed after two days of MN patch application in 15 out of 19 subjects for CMC-MN and in 18 out of 19 subjects for HN-MN (Figure 5D). These skin irritation observations are considered acceptable in the field of dermatology because the resulting ICDRG scores were the second-lowest levels (+?: doubtful reaction; faint erythema only) among the five levels of ICDRG scoring. Temporary erythema disappeared after seven days of MN patch application, and all cases reverted to their original skin condition (Figure 5D). These findings led us to conclude that both MN patches are safe to use as transcutaneous vaccine delivery devices without any serious adverse effects because all of the observed cases of skin irritation were dermatologically acceptable as slight and temporary local adverse reactions.

These results revealed that both novel faster-dissolving MN patches could reliably and safely puncture human skin and could deliver the loaded vaccine antigen into human skin with MN patch application. However, the MN patches need to be applied for at least 2 h to completely dissolve MNs in human skin. Hence, further optimization of MN formulation will be required to meet our target of MN dissolution in human skin within 30 min.

## 4. Discussion

Because solid MN and hollow MN have several limitations and issues for the practical use, many scientists are focusing on the features and advantages of dissolving MN and coated MN, and vigorously investigating their practical use for transcutaneous vaccination devices [11]. We also considered that the dissolving MN was the most promising for clinical use and demonstrated that MH, a dissolving MN, was clinically available for transcutaneous vaccine delivery and a feasible alternative vaccination method to conventional injections [14,15]. However, we identified potential issues through clinical researches during the MH development for clinical use. The current MH needs to be applied to human skin for approximately 6 h to achieve reliable delivery of the loaded vaccine antigen into human skin because of the slow dissolution of MNs in MH. In the present study, we set the goal of MN-dissolution kinetics in human skin within 30 min for novel faster-dissolving MN patches, based on the current clinical procedure for vaccination in Japan.

Both MNs in CMC-MN and HN-MN completely dissolved in rat skin after 5-min application and in human skin after 2-h application, respectively. These results are in line with our previous reports in which the MNs in MH could completely dissolve in mouse or rat skin after 1-h application and in human skin after 6-h application, suggesting that MN-dissolution kinetics in the skin differ according to the animal species [14,15]. The formulation design strategy for the dissolving MNs based on a single thickening agent represents an effective way to have the MN dissolve faster because the MN-dissolution kinetics in our novel MNs are superior to those in MH in both rats and humans. However, it is necessary to further improve the formulation in the current prototypes because the MNs could not completely dissolve within 30 min in human skin and thus did not meet our initial goal. The dissolution mechanism of the MNs in the skin is presumed to be as follows: MNs first hydrate, then gelate, and finally dissolve in the skin. Therefore, a hydration enhancer incorporated into MNs to accelerate gelation may be effective in improving the MN dissolution rate. Raphael et al. reported that their Dissolving Nanopatch applied CMC/sorbitol formulation (w/w ratios of 1:30 for CMC: sorbitol), had 100 μm long MNs in length and could release the loaded OVA within 5 s after mice earlap application [18]. Yan et al. reported that their Nanocomposite-strengthened dissolving MNs incorporating layered double hydroxides nanoparticles into the CMC-based MN formulation, which had 165 μm long MNs in length, had high MN failure force and rapid MN dissolution rate in pig skin, and released the payload within 1 min [22]. Taken together, incorporating sugar alcohol and layered double hydroxides nanoparticles might be an effective formulation strategy to make MNs dissolve faster.

Our results from the in vivo rat study using OVA-loaded MN patches revealed that TCI via our novel MN patches induced stronger antigen-specific immune responses compared to conventional SCI (Figure 4). As with our previous works [3,23], these strong primary immune responses result from the fact that MN can reliably and efficiently deliver vaccine antigen into the living epidermis and dermis, and prolong antigen deposition in the skin compared with ID injection (Figure 3). Therefore, TCI using MN is expected to induce strong antigen-specific immune responses because it is assumed that MN can efficiently deliver vaccine antigens to skin-resident APCs, and skin-resident APCs effectively induce acquired immune responses by increasing antigen capture efficiency of APCs.

In the present clinical research, no skin irritation was observed at the application sites of either MN patches. The results of clinical research also suggested that our novel faster-dissolving MN patches exhibit sufficient human skin puncturability because of the elevated TEWL associated with the formed micropores in the stratum corneum after skin application. Hence, we judged that the novel MN patches essentially have the same critical quality attributes as MH: safety and the reliable human skin puncturability. However, we consider that the current faster-dissolving MN formulation needs to be further improved to ensure robust skin puncturability because their MN failure force was approximately 20% of MH [14]. According to a report by Allender et al. presenting MNs of similar shape to ours (length: 750 μm, width at tip: 10 μm, width at basement: 200 μm, needle-to-needle spacing: 400 μm), CMC/maltose formulation (w/w ratios of 1:1 for CMC:maltose) could increase the MN failure force [17]. Sugar such as trehalose and maltose incorporated into the thickening agent (CMC and HN) may improve the MN failure force, however, the performance trade-off between MN failure force and rapid MN dissolution in the skin should be considered. To identify the ideal MN failure force in the improved MNs that can be completely dissolved in human skin within 30 min after application, we are going to further accumulate fundamental investigation results in consideration of conducting the additional clinical research.

## 5. Conclusions

We demonstrated that two types of novel faster-dissolving MN patches could reliably and safely puncture human skin and deliver the loaded vaccine antigen into human skin. Both CMC-MN and HN-MN patches shorten the skin application time in humans, and are promising as effective transcutaneous vaccine delivery devices for human use because both MN patches could strongly induce antigen-specific immune responses compared with the conventional SCI in rats.

## Figures and Tables

**Table 1:** Scoring of patch test in accordance with ICDRG.

Score	Reactions
−	Negative reaction
+?	Doubtful reaction; faint erythema only
+	Weak (non-vesicular) positive reaction; erythema, infiltration and possibly papules
++	Strong (vesicular) positive reaction; erythema, infiltration, papules, vesicles
+++	Extreme positive reaction; bullous reaction
IR	Irritant reaction
