# Correction: Development of Novel Faster-Dissolving Microneedle Patches for Transcutaneous Vaccine Delivery. *Pharmaceutics*, 2017, 9(3), 27

**DOI:** 10.3390/pharmaceutics9040059

**Published:** 2017-12-16

**Authors:** Akihiko Ono, Sayami Ito, Shun Sakagami, Hideo Asada, Mio Saito, Ying-Shu Quan, Fumio Kamiyama, Sachiko Hirobe, Naoki Okada

**Affiliations:** 1 Project for Vaccine and Immune Regulation, Graduate School of Pharmaceutical Sciences, Osaka University, 1-6 Yamadaoka, Suita, Osaka 565-0871, Japan; ono-ak@phs.osaka-u.ac.jp (A.O.); ito-sa@phs.osaka-u.ac.jp (S.I.); sakagami-s@phs.osaka-u.ac.jp (S.S.); 2 Laboratory of Biotechnology and Therapeutics, Graduate School of Pharmaceutical Sciences, Osaka University, 1-6 Yamadaoka, Suita, Osaka 565-0871, Japan; sachi-be@phs.osaka-u.ac.jp; 3 Department of Dermatology, Nara Medical University, 840 Shin-cho, Kashihara, Nara 634-8522, Japan; asadah@naramed-u.ac.jp; 4 CosMED Pharmaceutical Co. Ltd., 32 Higashikujokawanishi-cho, Minami-ku, Kyoto 601-8014, Japan; saito@cosmed-pharm.co.jp (M.S.); quan@cosmed-pharm.co.jp (Y.-S.Q.); kamiyama@cosmed-pharm.co.jp (F.K.); 5 Laboratory of Vaccine and Immune Regulation, Graduate School of Pharmaceutical Sciences, Osaka University, 1-6 Yamadaoka, Suita, Osaka 565-0871, Japan

The authors wish to make a change to their published paper [1]. The unit in the *y* axis of Figure 5C should be changed from “%” to “g/h·m^2^”. Therefore, we wish to replace the figure with the correct one below (Figure 1C). 

The authors apologize for any inconvenience caused. The changes do not affect the scientific results. The manuscript will be updated and the original will remain online on the article webpage (http://www.mdpi.com/1999-4923/9/3/27), with a reference to this addendum.

## Figures and Tables

**Figure 1:**
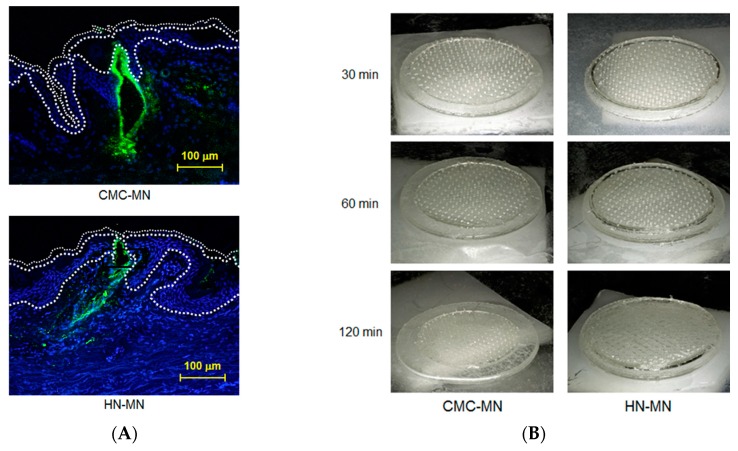

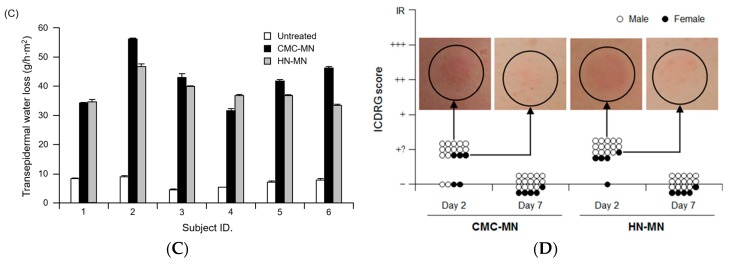
Antigen delivery images, dissolution of MNs, skin puncturability, and safety using faster-dissolving MN patches on human skin. (**A**) F-OVA-loaded MN patches were applied to the resected human dermal tissues. In the fluorescence images, the area between the top line and the middle line represents the stratum corneum, the area between the middle line and the bottom line represents the living epidermis, and the dermis is located under the bottom line. Green and blue fluorescence indicate F-OVA and nucleus (DAPI), respectively; (**B**) Placebo CMC-MN and HN-MN patches were each applied to the skin of upper outer arm of 19 healthy volunteers (14 male and five female) for the indicated times. After removal of MN patches, the remaining MNs on each CMC-MN and HN-MN patch were photographed using a stereoscopic microscope; (**C**) After MN patch applications of 30 min, TEWL at the application site was measured immediately after MN patch removal. Data are expressed as mean ± SD of results from three measurements; (**D**) Skin irritation caused by application of MN patches was assessed in accordance with the ICDRG score. Each plot expresses the score of an individual subject. Four photographs show the site judged as +? “doubtful reaction; faint erythema only.”

## References

[1] Ono A., Ito S., Sakagami S., Asada H., Saito M., Quan Y.S., Kamiyama F., Hirobe S., Okada N. (2017). Development of Novel Faster-Dissolving Microneedle Patches for Transcutaneous Vaccine Delivery. Pharmaceutics.

